# A novel multivariate logistic model for predicting risk factors of failed treatment with carbapenem-resistant *Acinetobacter baumannii* ventilator-associated pneumonia

**DOI:** 10.3389/fpubh.2024.1385118

**Published:** 2024-05-09

**Authors:** Ke Sun, Fangchen Peng, Kaiqiang Xu, Yong Liu, Xuanping Zhou, Nan Shang, Chao Li

**Affiliations:** ^1^Department of Pharmacy, The First Hospital of Shanxi Medical University, Taiyuan, China; ^2^Shanxi Province People’s Hospital, Taiyuan, China; ^3^Qinhuangdao Center for Disease Control and Prevention, Qinhuangdao, Hebei, China; ^4^Shandong Public Health Clinical Center, Jinan, Shangdong, China; ^5^School of Pharmacy, Shanxi Medical University, Taiyuan, China

**Keywords:** hospital-acquired infection, carbapenem-resistant *Acinetobacter baumannii* ventilator-associated pneumonia, tigecycline, multivariate logistic model, predictive model, nomogram model

## Abstract

**Background:**

This study aimed to explore the risk factors for failed treatment of carbapenem-resistant *Acinetobacter baumannii* ventilator-associated pneumonia (CRAB-VAP) with tigecycline and to establish a predictive model to predict the incidence of failed treatment and the prognosis of CRAB-VAP.

**Methods:**

A total of 189 CRAB-VAP patients were included in the safety analysis set from two Grade 3 A national-level hospitals between 1 January 2022 and 31 December 2022. The risk factors for failed treatment with CRAB-VAP were identified using univariate analysis, multivariate logistic analysis, and an independent nomogram to show the results.

**Results:**

Of the 189 patients, 106 (56.1%) patients were in the successful treatment group, and 83 (43.9%) patients were in the failed treatment group. The multivariate logistic model analysis showed that age (OR = 1.04, 95% CI: 1.02, 1.07, *p* = 0.001), yes. of hypoproteinemia (OR = 2.43, 95% CI: 1.20, 4.90, *p* = 0.013), the daily dose of 200 mg (OR = 2.31, 95% CI: 1.07, 5.00, *p* = 0.034), yes. of medication within 14 days prior to surgical intervention (OR = 2.98, 95% CI: 1.19, 7.44, *p* = 0.019), and no. of microbial clearance (OR = 0.31, 95% CI: 0.14, 0.70, *p* = 0.005) were risk factors for the failure of tigecycline treatment. Receiver operating characteristic (ROC) analysis showed that the AUC area of the prediction model was 0.745 (0.675–0.815), and the decision curve analysis (DCA) showed that the model was effective in clinical practice.

**Conclusion:**

Age, hypoproteinemia, daily dose, medication within 14 days prior to surgical intervention, and microbial clearance are all significant risk factors for failed treatment with CRAB-VAP, with the nomogram model indicating that high age was the most important factor. Because the failure rate of CRAB-VAP treatment with tigecycline was high, this prediction model can help doctors correct or avoid risk factors during clinical treatment.

## 1 Introduction

*Acinetobacter baumannii* is a significant opportunistic pathogen widely present in medical environments, capable of causing severe nosocomial infections (1). Prolonged and excessive use of carbapenem antibiotics, such as imipenem and meropenem, exposes bacteria to high drug concentrations, leading to the emergence of drug-resistant strains. The transmission of carbapenem-resistant *Acinetobacter baumannii* (CRAB) in healthcare settings is facilitated by the spread of resistance genes between bacteria and inadequate infection control measures in hospitals (2, 3). The rates of resistance of *Acinetobacter baumannii* to meropenem and imipenem increased from 30.1 and 39.0% in 2005 to 71.5 and 72.3% in 2021, respectively, with the detection rate of CRAB gradually increased (4). Recent studies demonstrated that CRAB has the highest detection rate in the respiratory tract (60 ~ 87%), especially in ventilator-associated pneumonia (VAP) (5, 6).

VAP ranks among the most common nosocomial infections in the intensive care unit (ICU), contributing to increased mortality rates and healthcare expenditures, which were found to be associated with the delayed recognition and treatment of VAP due to drug-resistant *A. baumannii* (1, 7). Some studies have found that CRAB-VAP is not only closely associated with patients’ clinical outcomes (such as length of hospital stay and treatment costs) but also significantly correlated with patients’ prognosis (such as mortality rate and incidence of complications) (8, 9). Therefore, the treatment strategy for CRAB-VAP is particularly important.

In 2023, the Infectious Diseases Society of America (IDSA) guidelines recommended medications for treating CRAB, including ampicillin-sulbactam, polymyxins, and tetracycline derivatives. Tigecycline, as one of the few antibiotics effective against CRAB, was a crucial component of treatment regimens, especially when patients have concurrent renal insufficiency or when certain medications are unavailable (such as the intravenous formulation of minocycline, not marketed in China) (10–13). However, a study has shown that compared to other antimicrobial drugs, the use of tigecycline in treating CRAB-VAP increases the risk of patient mortality, leading to controversy over its efficacy and suggesting that it may not be suitable for treating CRAB-VAP (14). Therefore, investigating high-risk populations for tigecycline treatment failure in CRAB-VAP is imperative to select more suitable alternatives early in the treatment and reduce the likelihood of treatment failure.

Hence, this study conducted a multicenter retrospective study to evaluate the clinical characteristics and risk factors of CRAB-VAP patients who failed treatment with tigecycline and constructed a nomogram model of risk factors for treatment failure with a view to providing clinical diagnosis and treatment.

## 2 Methods

### 2.1 Study cohort and route

**Figure 1:**
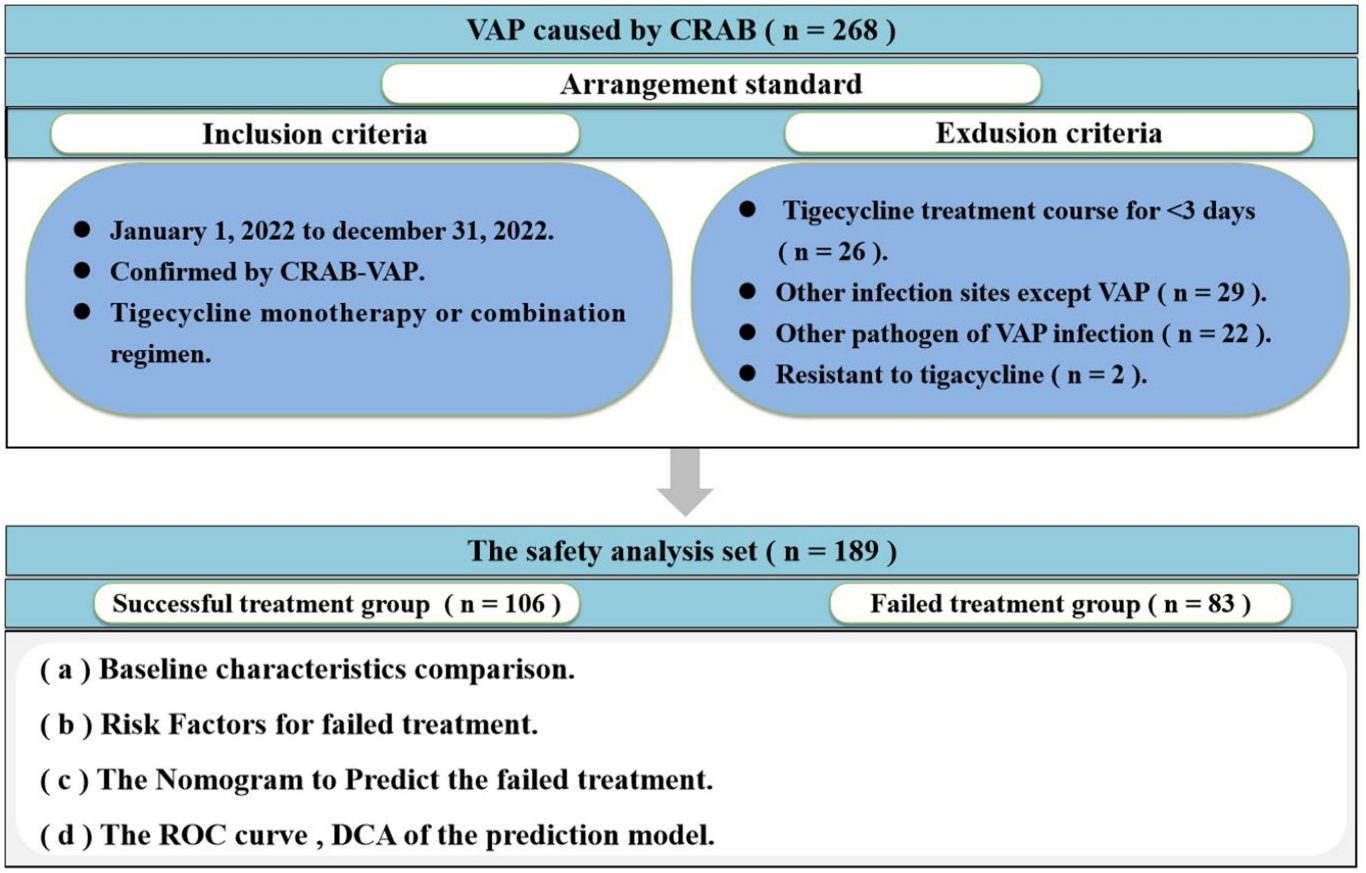
Flowchart of the case selection process.

This multicenter, retrospective cohort study was carried out at The First Hospital of Shanxi Medical University, a 2,504-bed Grade 3 A National-level hospital, and Shanxi Provincial People’s Hospital, a 2,584-bed Grade 3 A National-level hospital. The study focused on patients with CRAB-VAP between 1 January 2022 and 31 December 2022. The inclusion criteria were as follows: (i) patients confirmed by CRAB-VAP and (ii) anti-infective treatment with tigecycline monotherapy or combination regimen. The exclusion criteria were as follows: (i) patients less than 18 years and (ii) tigecycline treatment course for <3 days. Only the first CRAB-VAP was included if there were multiple repeated during the study period. Patient demographics (sex, age, height, weight, and BMI), basic disease, predrug patients Sequential Organ Failure Assessment (SOFA), clinical and microbiological data (blood routine, procalcitonin, and drug sensitivity results), drug information (drug time, drug dose, and treatment), and other relevant information were obtained from the hospitals’ electronic medical record systems. The flow chart of this study is shown in Figure 1. The clinical efficacy of patients was used as a treatment outcome.

### 2.2 Definitions

CRAB was defined as *Acinetobacter baumannii* strains resistant to imipenem and meropenem (15). Hospital-acquired lung infection (HAP) was defined as patients not receiving invasive mechanical ventilation during hospitalization and were not in the latent period of pathogenic infection, while the onset of new pneumonia occurred 48 h after admission. VAP was defined as pneumonia occurring within 48 h after mechanical ventilation in patients undergoing endotracheal intubation or tracheotomy and within 48 h after withdrawal or extubation on mechanical ventilation (16, 17). The microbial clearance was defined as no original pathogenic bacteria cultured from the original infection site specimens after treatment, or symptoms and signs of infection disappeared, or culture specimens were not obtained. Microbial non-clearance was defined as primary pathogenic bacteria cultured from the original infection site after treatment (18).

CRAB causes pneumonia as follows: (i) patients had signs of bacterial infection (fever, white blood cells increased, neutrophils increased, PCT, or C-reactive protein increased), (ii) patients had the clinical symptoms consistent with pneumonia and radiographic appearance of new, or persistent, or aggravated pulmonary exudation, infiltration, and consolidation, (iii) patients had high-risk factors for resistant bacterial infection (such as basic disease, immune status, prior antimicrobial use, and other risk factors associated with morbidity), (iv) the specimen collection was qualified and the sputum smear showed coccobacillus engulfed by leukocytes, and (v) more than two sputum cultures showed the growth of pure *A. baumannii* or the dominant growth of *A. baumannii* (19).

The treatment combination regimen was defined as two or more antimicrobial agents used for treating CRAB, and the combination lasted greater than 72 h.

Successful treatment was defined as patients’ clinical characteristics returning to normal or having a significant improvement, and no new anti-infection regimen or surgical treatment is required for the initial infection of CRAB. The failed treatment was defined as initial signs of infection persisting after 72 h of tigecycline treatment, changes in antibiotic therapy or surgical intervention, or initial signs of infection reappearing (20).

### 2.3 Pathogen identification and drug susceptibility testing

Pathogen identification and drug susceptibility testing used an automated microbial identification and drug susceptibility analysis system (Moliere, France). We used the European Committee for Antimicrobial Susceptibility Testing (EUCAST) breakpoints, tigecycline of minimum inhibitory concentration (MIC) for A. *baumannii* ≤ 2 was considered to be sensitive (21, 22).

### 2.4 Statistical analysis

Continuous variables were expressed as mean ± standard deviation (mean ± sd) or median (IQR). Categorical variables were expressed as numbers with percentages [*n* (%)]. For continuous variables, a *t*-test or Mann–Whitney *U*-test was used to assess for normality and analysis. For categorical variables, the chi-square test or two-tailed Fisher’s exact test was used to compare between groups. The strength of associations was assessed in terms of the odds ratio (OR) and 95% confidence interval (CI). Univariate regression analysis used logistic regression analysis was performed after further screening for variables with a *p*-value of <0.1 in the univariate to determine independent diagnosis factors of failed treatment. In addition, this study established an independent nomogram based on risk factors to predict the probability of failed treatment. The receiver operating characteristic (ROC) curves were used to evaluate the accuracy of the nomograms. The discrimination of the nomogram was verified using a calibration plot with 1,000 bootstrap samples. The decision curve analysis (DCA) was a method to evaluate the clinical utility of the predictive model. A *p*-value of 0.05 was taken as the nominal level to determine the statistical significance of all analyses. The missing data in this study were very limited; individual patients lacked information on comorbid chronic diseases. Data analysis was performed in R (version 4.1.3).

## 3 Results

### 3.1 A comparison of baseline characteristics among 189 patients with CRAB-VAP

Overall, 268 patients were enrolled, of whom 189 were included in the safety analysis set (Figure 1). A total of 79 patients were excluded from the final analysis set due to the tigecycline treatment course for <3 days (*n* = 26), other infection sites except VAP (*n* = 29), other pathogens of VAP infection (*n* = 22), and resistance to tigecycline (*n* = 2). Data from the safety analysis set were collected from 1 January 2022 to 31 December 2022.

The baseline characteristics of 189 patients with CRAB-VAP are listed in Table 1. The median age of patients was 62 (53 ~ 71) years, and 128 (67.7%) of them were male, with 106 patients in the successful treatment group and 83 patients in the failed treatment group. Variables such as age, sex, BMI, hypertension, diabetes, CAD, hyperlipemia, liver dysfunction, usage within 48 h of incubation, treatment duration exceeding 7 days, combination, and medication within 14 days prior to surgical intervention were not different between the two groups. The study found that the failed treatment group had a higher usage of immunosuppressants, proportion of hypoproteinemia, daily dose of 200 mg, and proportion of patients with SOFA≥7 (*p* < 0.05). In the successful treatment group, there was a higher proportion of administering a combination, daily dose of 100 mg, and microbial clearance (*p* < 0.05). In this study, there was a variety of combination therapy regimens, which precluded statistical analysis. Common combination regimens included the following: tigecycline with sulbactam preparations, tigecycline with colistin, tigecycline with meropenem, tigecycline with aminoglycoside antibiotics, and tigecycline with polymyxins.

**Table 1:** A comparison of baseline characteristics in the study population regarding treatment success.

	Total (*n* = 189)	Failed treatment group (*n* = 83)	Successful treatment group (*n* = 106)	*p* value
Age, year	62.0 [53.0; 71.0]	64.0 [54.5; 74.5]	58.0 [50.5; 69.0]	0.051
Sex				0.823
Female	61 (32.3)	28 (33.7)	33 (31.1)	
Male	128 (67.7)	55 (66.3)	73 (68.9)	
BMI, kg/m^2^	23.4 [21.3; 25.4]	24.0 [21.2; 26.9]	22.9 [21.3; 24.9]	0.125
Hypertension	55 (29.1)	20 (24.1)	35 (33.0)	0.238
Diabetes	40 (21.2)	20 (24.1)	20 (18.9)	0.488
CAD	21 (11.1)	10 (12.0)	11 (10.4)	0.897
Hyperlipemia	6 (3.17)	5 (6.02)	1 (0.94)	0.088
Liver dysfunction	30 (15.9)	17 (20. 5)	13 (12.3)	0.182
Usage of immunosuppressant	55 (29.1)	31 (37.3)	24 (22.6)	0.041
Hypoproteinemia	105 (55.6)	57 (68.7)	48 (45.3)	0.002
Usage within 48 h of incubation	133 (70.4)	60 (72.3)	73 (68.9)	0.726
Administering a combination	97 (51.3)	35 (42.2)	62 (58.5)	0.037
Daily dose				0.026
100 mg	63 (33.3)	20 (24.1)	43 (40.6)	
200 mg	126 (66.7)	63 (75.9)	63 (59.4)	
Treatment duration exceeds 7 days	117 (61.9)	48 (57.8)	69 (65.1)	0.385
Combination	155 (82.0)	69 (83.1)	86 (81.1)	0.869
SOFA ≥7	40 (21.2)	24 (28.9)	16 (15.1)	0.033
Medication within 14 days Prior to surgical intervention	41 (21.7)	23 (27.7)	18 (17.0)	0.110
Microbial clearance	50 (26.6)	13 (15.7)	37 (35.2)	0.004

BMI, body mass index; CAD, coronary artery disease; SOFA, sequential organ failure assessment.

### 3.2 Univariate logistic regression analysis for failed treatment

As shown in Table 2, univariate logistic analysis results showed that age, BMI, usage of immunosuppressants, hypoproteinemia, administering a combination, daily dose, SOFA≥7, medication within 14 days prior to surgical intervention, and microbial clearance were associated with failed treatment.

**Table 2:** Univariate logistic regression analysis for failed treatment.

Variable	OR (95% CI)	*p*-value
Age, year	1.02 (1.00–1.04)	0.084
Sex		
Female	Reference	
Male	0.89 (0.48–1.64)	0.704
BMI, kg/m^2^	1.08 (1.01–1.17)	0.048
Hypertension		
No	Reference	
Yes	0.64 (0.34–1.23)	0.182
Diabetes		
No	Reference	
Yes	1.37 (0.68–2.75)	0.383
CAD		
No	Reference	
Yes	1.18 (0.48–2.94)	0.717
Hyperlipemia		
No	Reference	
Yes	6.73 (0.77–58.77)	0.085
Liver dysfunction		
No	Reference	
Yes	1.84 (0.84–4.05)	0.128
Usage of immunosuppressant		
No	Reference	
Yes	2.04 (1.08–3.85)	0.028
Hypoproteinemia		
No	Reference	
Yes	2.65 (1.45–4.83)	0.001
Usage within 48 h of incubation		
No	Reference	
Yes	1.18 (0.63–2.22)	0.609
Administering a combination dose		
No	Reference	
Yes	0.52 (0.29–0.93)	0.027
Daily dose		
100 mg	Reference	
200 mg	2.15 (1.14–4.06)	0.018
Treatment duration exceeds 7 days		
No	Reference	
Yes	0.74 (0.41–1.33)	0.308
Combination		
No	Reference	
Yes	1.15 (0.54–2.43)	0.722
SOFA ≥7		
No	Reference	
Yes	2.29 (1.12–4.67)	0.023
Surgical intervention within 14 days prior to medication administration		
No	Reference	
Yes	1.87 (0.93–3.77)	0.078
Microbial clearance		
No	Reference	
Yes	0.34 (0.17–0.70)	0.003

BMI, body mass index; CAD, coronary artery disease; SOFA, sequential organ failure assessment, OR, odds ratio; CI, confidence interval.

### 3.3 Multivariate logistic regression analysis for failed treatment with CRAB-VAP

Table 3 summarizes the results in the multivariate logistic model. The result showed that age, hypoproteinemia, daily dose, medication within 14 days prior to surgical intervention, and microbial clearance were significant determinants among all the factors included. Every 1-year increase in age corresponded to 1.04 (95% CI: 1.02, 1.07) in failed treatment. Compared with the no. of hypoproteinemia level, individuals with yes. of hypoproteinemia level had 2.43 (95% CI: 1.20, 4.90). Compared with a daily dose of 100 mg, individuals with a 200 mg level had 2.31 (95% CI: 1.07, 5.00). Compared with no. of medication within 14 days prior to surgical intervention level, individuals with the yes level had 2.98 (95% CI: 1.19, 7.44). Compared with the no. of microbial clearance level, individuals with the yes level had 0.31 (95% CI: 0.14, 0.70).

**Table 3:** Multivariate logistic regression analysis for failed treatment.

Variable	OR (95% CI)	*p* value
Age, year	1.04 (1.02–1.07)	0.001
BMI, kg/m^2^	1.03 (0.94–1.12)	0.57
Hyperlipemia		
No	Reference	
Yes	9.70 (0.86–109.13)	0.066
The usage of immunosuppressant		
No	Reference	
Yes	2.17 (0.96–4.91)	0.062
Hypoproteinemia		
No	Reference	
Yes	2.43 (1.20–4.90)	0.013
Administering a combination dose		
No	Reference	
Yes	0.78 (0.37–1.64)	0.505
Daily dose		
100 mg	Reference	
200 mg	2.3 1 (1.07–5.00)	0.034
SOFA ≥7		
No	Reference	
Yes	1.97 (0.86–4.51)	0.108
Medication within 14 days Prior to surgical intervention		
No	Reference	
Yes	2.98 (1.19–7.44)	0.019
Microbial clearance		
No	Reference	
Yes	0.31 (0.14–0.70)	0.005

BMI, body mass index; SOFA, sequential organ failure assessment, OR, odds ratio; CI, confidence interval; SE, standard error.

### 3.4 The nomogram to predict the failed treatment with CRAB-VAP

**Figure 2:**
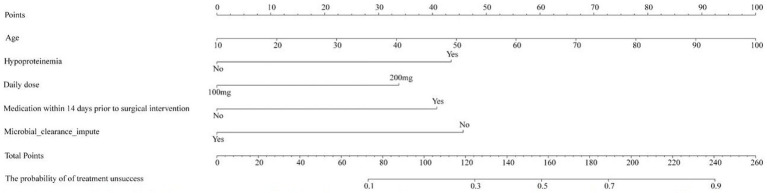
The nomogram model to predict the failed treatment with carbapenem-resistant *Acinetobacter baumannii* ventilator-associated pneumonia.

**Figure 3:**
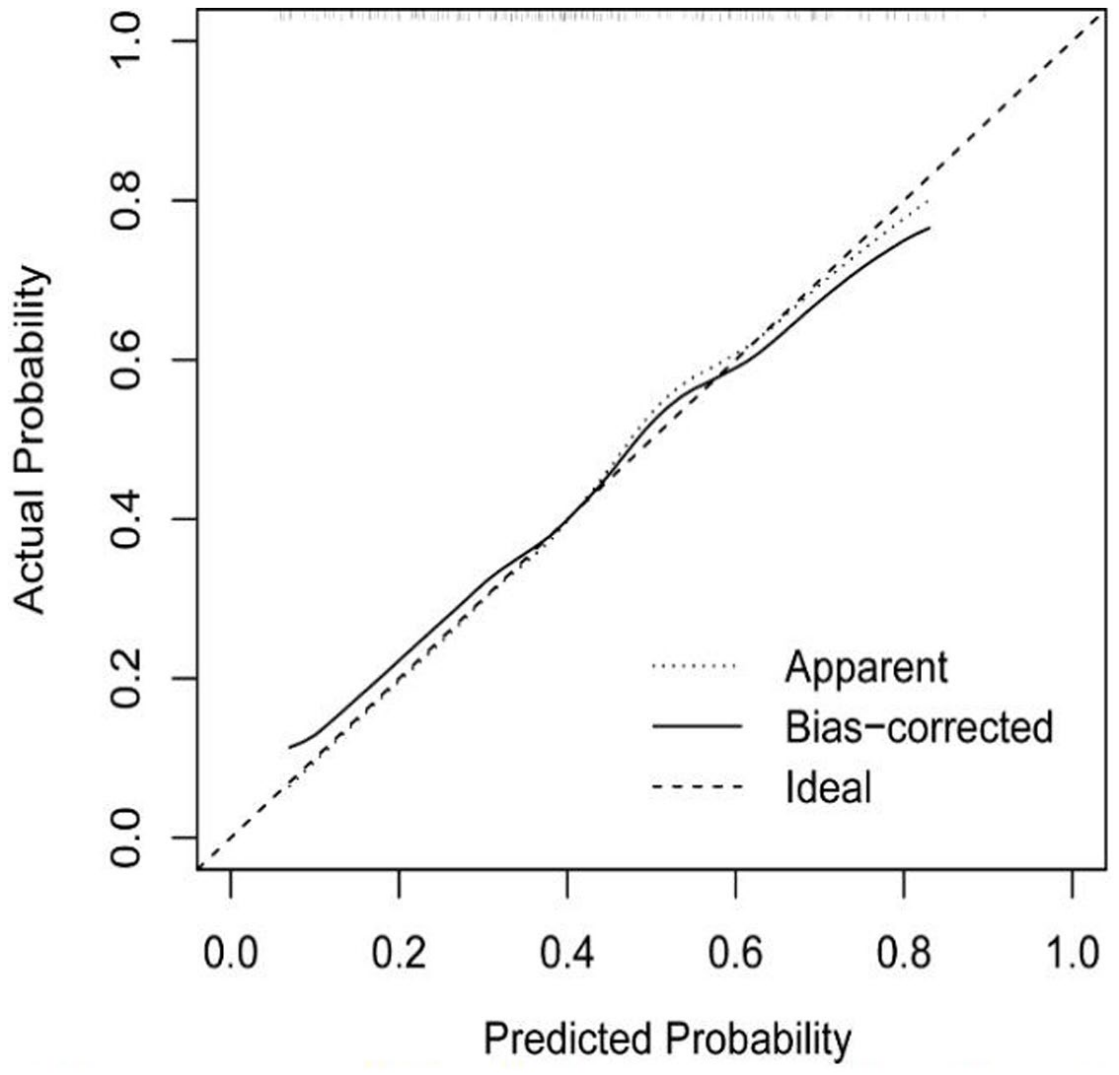
The calibration curve of the failed treatment with carbapenem-resistant *Acinetobacter baumannii* ventilator-associated pneumonia prediction model.

**Figure 4:**
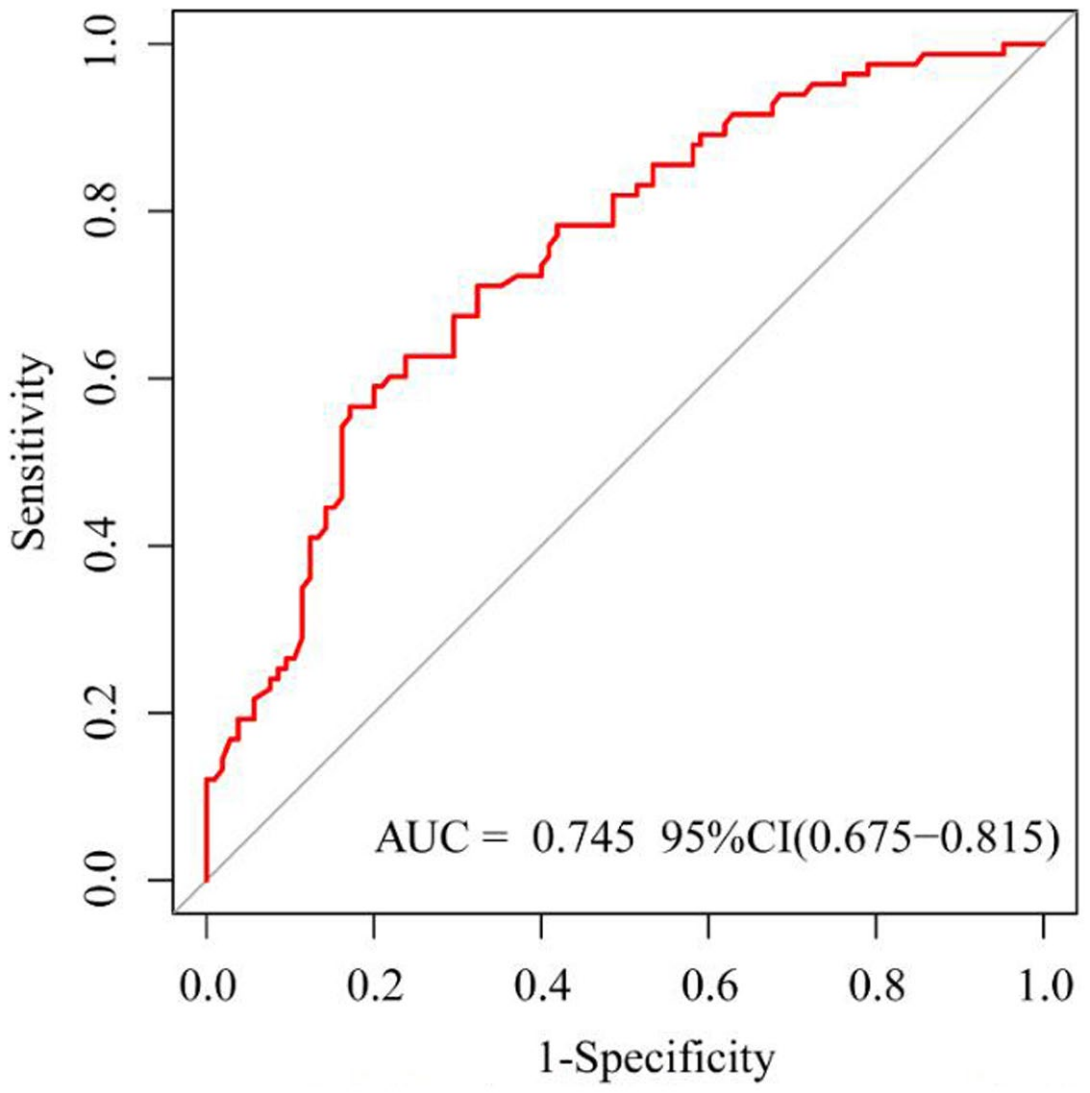
The receiver operating characteristic curve of the failed treatment with carbapenem-resistant *Acinetobacter baumannii* ventilator-associated pneumonia prediction model.

**Figure 5:**
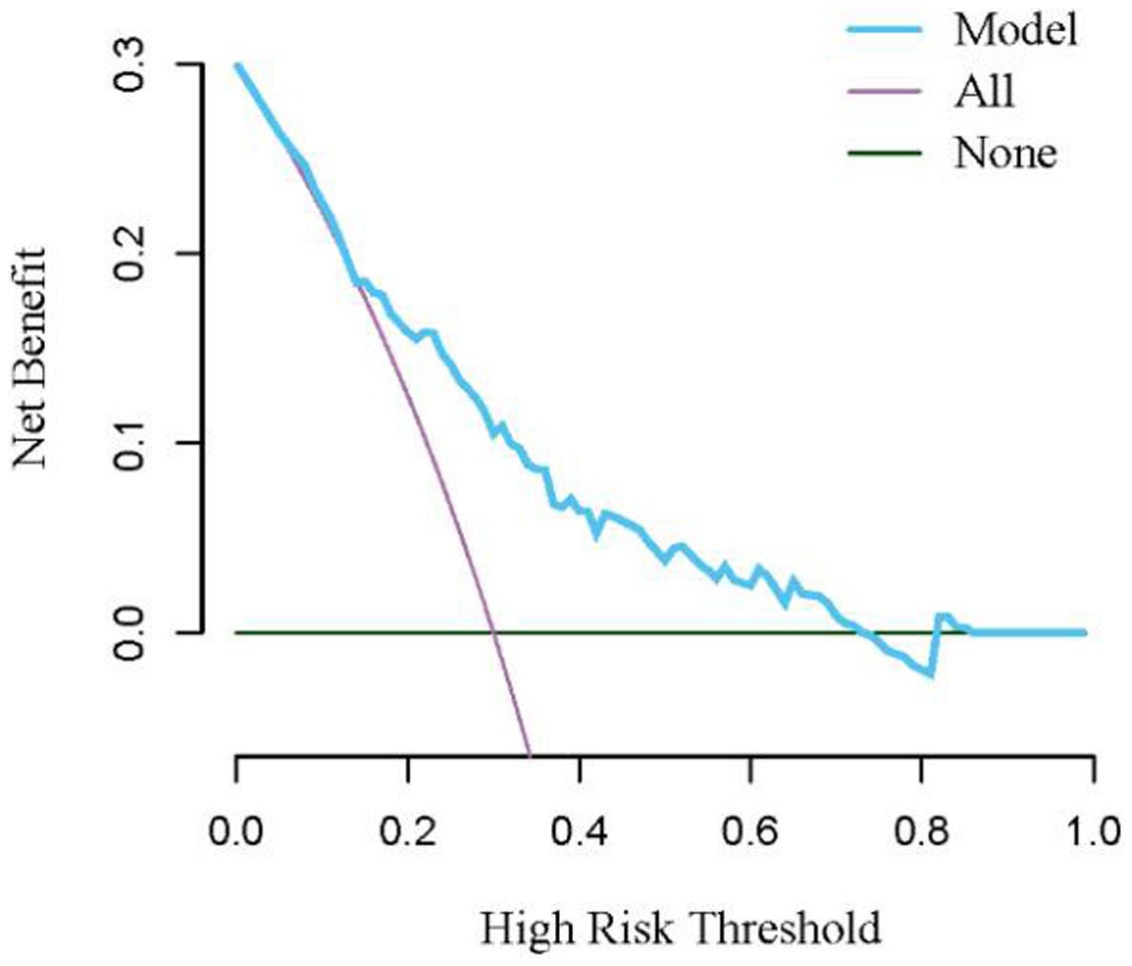
The decision curve analysis for the nomogram model for the failed treatment with carbapenem-resistant *Acinetobacter baumannii* ventilator-associated pneumonia prediction model.

Based on risk factors determined by multivariable logistic regression, the study constructed a nomogram to predict the failed treatment with CRAB-VAP (Figure 2). Age, yes. of hypoproteinemia, a daily dose of 200 mg, yes. of medication within 14 days prior to surgical intervention, and no. of microbial clearance were risk factors for the failure of tigecycline treatment. To prevent deviations in the results, a constructed calibration curve was used in this study (Figure 3). The calibration curve using the bootstrap method (1,000 times) was plotted, which showed a good agreement between the predicted model and the actual observations. The ROC analysis revealed that the AUC value of the nomogram to predict the failed treatment with CRAB-VAP reached 0.745 (0.675–0.815), indicating that the model had good discrimination ability (Figure 4). Additionally, DCA showed that the nomogram model was effective in clinical practice (Figure 5).

## 4 Discussion

CRAB is widely found in medical environments and has a high tolerance, presenting a great challenge to healthcare around the world. In 2019, the Centers for Disease Control and Prevention listed CRAB as an emergency threat in reporting antibiotic resistance threats (23). Therefore, the therapeutic strategies for CRAB-VAP have attracted much attention (11, 24). The use of tigecycline as a broad-spectrum antibiotic in the treatment of CRAB-VAP has indeed sparked a debate. On the one hand, certain studies suggest its effectiveness for some CRAB-VAP patients, offering them a useful treatment option. However, on the other hand, some research also indicates poor outcomes with tigecycline therapy for CRAB-VAP, potentially leading to increased overall mortality rates (10). This study differs from previous research in that it does not compare tigecycline with other antibacterial drugs to observe its efficacy and safety in CRAB treatment. Instead, it introduces a novel therapeutic approach: selecting appropriate drugs based on patients’ clinical characteristics to offer a new perspective for CRAB treatment.

In this study, 189 patients were finally included, of which 106 patients were successfully treated, and the treatment of 83 patients failed. The CRAB-VAP failure rate caused by tigecycline treatment was high at 43.9%, which was consistent with the results of previous studies. Several risk factors had been linked to the failed treatment with CRAB-VAP, such as age, hypoproteinemia, and surgical intervention 14 days before treatment, which was homogeneous with this study’s results. As the patients get older, body function gradually degenerates, organ function and immunity also decline, and the compensatory ability to tolerate infection decreases, resulting in an increased risk of anti-infection treatment failure (25). Protein is one of the important components of the body, involved in the body’s metabolism and cell regeneration. Hypoproteinemia can lead to malnutrition in patients, which can affect the treatment of infections (26). In addition, tigecycline is a high-protein-binding drug, which can lead to an increase in free drugs in the body, an increase in drug clearance, reduced blood concentration, and then affect the anti-infection effect (27). Patients experience a local inflammatory response during surgical procedures to cut and manage trauma, and long-term surgical operations would also consume energy and nutrition, putting patients in a semi-healthy state and reducing the body’s ability to resist infection (28).

Furthermore, this study found that no. of microbial clearance was an independent risk factor for the failure of tigecycline treatment. CRAB is known to be extremely resistant, often presenting as multidrug resistance or pan-drug resistance (29). Current treatment options are very limited and cannot completely kill CRAB (30, 31). Therefore, current treatment regimens usually only use effective antimicrobial drugs to control the replication and growth of pathogens rather than completely remove them, and many clinical studies also take the disappearance of clinical infection manifestations as a clinical outcome. However, this study found that CRAB clearance might have an important impact on treatment outcomes (32). Patients who failed treatment with CRAB-VAP should choose bactericidal antimicrobial agents during treatment as a part of the treatment regimen.

In this study, it was unexpectedly found that a daily dose of 200 mg of tigecycline was an independent risk factor for treatment failure compared to a daily dose of 100 mg. In a study, it was demonstrated that for susceptible CRAB strains (MIC ≤0.5 mcg/ml), daily administration of 200 mg of tigecycline can increase the drug’s concentration in plasma and lungs. However, the article also indicates that as the MIC increases to MIC ≥1 mcg/ml, less than 10% of patients can achieve effective treatment (33). This highlights the significant impact of the MIC values of CRAB strains on treatment outcomes. The use of tigecycline in CRAB infections has led to a shift in its MIC values. Research indicates that from 2016 to 2021, the MIC of *A. baumannii* isolates against tigecycline increased from 1 mcg/ml to 2 mcg/ml, with a more significant increase in Asia (34). Elevated MIC levels pose a risk for tigecycline treatment failure. Studies suggest that when the MIC is greater than 2, this drug should not be selected for treating CRAB infection (35, 36). Monte Carlo simulation results demonstrate that with the standard tigecycline regimen (100 mg loading dose, 50 mg maintenance dose, 12 h), the probability of target attainment (PTA) is 72 and 11% when the MIC is 1 mcg/ml and 2 mcg/ml, respectively. However, doubling the dosage to 100 mg every 12 h increases the corresponding PTA values to 99 and 71% (37). Therefore, it is recommended to double the dosage when the tigecycline MIC against *A. baumannii* is 2 mcg/ml (22). The patients included in this study had MIC ≤1 mcg/ml and MIC = 2 mcg/ml. In clinical practice, patients with MIC = 2 mcg/ml are more likely to receive a daily dose of 200 mg. Consequently, a daily dose of 200 mg increases the risk of treatment failure and may be more relevant to the population using this dose, particularly those with large MIC values. Unfortunately, due to the relatively small number of patients with MIC ≤1 mcg/ml in this study, the relationship between MIC and treatment failure cannot be definitively clarified.

Additionally, this study showed that the timing of medication administration, combination therapy regimen, the severity of organ dysfunction, and the duration of drug therapy do not significantly affect treatment outcomes. Previous studies have indicated that combined antimicrobial therapy is recommended for CRAB infection, and long-term treatment can reduce the 30-day mortality rate (10, 38). However, this study indicated no significant differences in these factors between the two groups.

This study was characterized by the analysis of the effect of the patient dosing regimen on the failed treatment with CRAB-VAP, which was conducive to the reasonable selection of antimicrobial drugs for CRAB. This study had several limitations, which include a small sample size, a lack of information on laboratory test indicators, and the limitations of the study area. These factors may have biased the study. Future prospective studies need to address these limitations.

## 5 Conclusion

This study showed that age, yes. of hypoproteinemia, a daily dose of 200 mg, yes. of medication within 14 days prior to surgical intervention, and no. of microbial clearance were significant risk factors for the failed treatment with CRAB-VAP. Additionally, although this study did not demonstrate the relationship between MIC and treatment outcomes, MIC variation may significantly affect the outcome of tigecycline therapy. The AUC area showed this predictive nomogram had good discrimination performance. This prediction model can help doctors predict factors of failed treatment with CRAB-VAP, and correct or avoid risk factors in clinical treatment, but the results should be based on the clinical experience of doctors and other auxiliary examinations.

## Data availability statement

The original contributions presented in the study are included in the article/Supplementary material, further inquiries can be directed to the corresponding author.

## Ethics statement

The studies involving humans were approved by Ethics Committee of First Hospital of Shanxi Medical University. The studies were conducted in accordance with the local legislation and institutional requirements. Written informed consent for participation was not required from the participants or the participants’ legal guardians/next of kin in accordance with the national legislation and institutional requirements.

## Author contributions

KS: Data curation, Methodology, Project administration, Supervision, Writing – original draft, Writing – review & editing. FP: Data curation, Writing – review & editing. KX: Writing – review & editing. YL: Writing – original draft. XZ: Data curation, Writing – review & editing. NS: Data curation, Writing – review & editing. CL: Data curation, Writing – review & editing.
